# The Current Status of the Alternative Use to Antibiotics in Poultry Production: An African Perspective

**DOI:** 10.3390/antibiotics9090594

**Published:** 2020-09-11

**Authors:** Letlhogonolo Andrew Selaledi, Zahra Mohammed Hassan, Tlou Grace Manyelo, Monnye Mabelebele

**Affiliations:** 1 Department of Agriculture and Animal Health, College of Agriculture and Environmental Science, University of South Africa, Florida 1710, South Africa; laselaledi@zoology.up.ac.za (L.A.S.); zahrabattal@gmail.com (Z.M.H.); manyelo.t.g@gmail.com (T.G.M.); 2 Department of Zoology and Entomology, Mammal Research Institute, Faculty of Natural and Agricultural Sciences, University of Pretoria, Hatfield 0028, South Africa; 3 Department of Agricultural Economics and Animal Production, University of Limpopo, Sovenga 0727, South Africa

**Keywords:** antimicrobial-resistance, prebiotic, probiotic, poultry, salmonella, tetracycline

## Abstract

Over the years the growth and health of food-producing animals have been enhanced by the use of antibiotics. These have helped reduce on-farm mortalities, lower incidences of diseases and more importantly improve productivity. Generally, the utilization of antibiotics in feed has been reevaluated since bacterial pathogens have established and shared a variety of antibiotic resistance mechanisms that can easily be spread within microbial communities. Multiple countries have introduced bans or severe restrictions on the non-therapeutic use of antibiotics. This has therefore warranted the urgent need for alternatives. Africa is facing its own challenges as it has been reported to be one of the continents with the highest number of foodborne diseases with approximately 91 million related diseases and 137,000 death per annum. Stakeholder and policy direction has been put in place to curb this escalation; however, the problem persists. The use of alternatives has been recommended and some successfully used in other countries as well as Africa, including pro- and prebiotics and phytochemicals. This then leads to the core aim of this review which is to (1) determine the extent to which antimicrobial-resistant pathogens have affected Africa, (2) assess the current measures put in place by Africa to reduces antimicrobial resistance and finally (3) explore the alternative use of antibiotics in poultry production. Improved sanitary conditions and farm biosecurity are important alternatives that could be adopted by farmers instead of depending on antibiotic drugs for disease control and prevention.

## 1. Introduction

The use of antibiotics in the poultry sector is mainly for treatment, prophylaxis and growth promotion. In many parts of the world, food-producing animals are given antibiotics daily to make them grow faster and prevent diseases [1]. This trend is likely to continue given the growing demand for the protein of animal origin. When antibiotics are used for the purposes of growth promotion a small amount is often administered as compared to therapeutic use. Therefore, this may cause bacteria to develop resistance to antibiotics [2]. The emergence and spread of antibiotic resistance compromise the nutritional and economic potential of poultry and other food-producing animals. This is a global concern that affects both animal and human ecosystems. According to the report commissioned by the United Kingdom (UK), it is estimated that almost 10 million people could die of bacteria that are resistant to the antibiotic by 2050 [3]. In the United States, over 2 million people get infected by antibiotic-resistance bacteria and around 23,000 of them die due to the resistance to treatment. The World Health Organization (WHO) has published a report regarding the incidence of antibiotic-resistance which shows an increase in the Asian continent [2]. In the US and Europe alone, antimicrobial-resistant cause overs 50,000 deaths annually [4]. Antimicrobial resistance threatens food security, animal welfare, longer treatment cycle and public health worldwide. There are many factors that contribute to the irrational use of antibiotic: Attitudes, perception of policymaker’s knowledge, manufacturer, prescribers, consumers and dispensers [4]. The European Union (EU) banned antibiotic use in animal production in 2006 [3]. A retrospective study analyzing the relationship between prior antibiotic use with antimicrobial-resistant was conducted in Indonesia and the results showed that patients who have a history of antibiotic use over the previous three months had shown an escalation of the probability of higher resistance matched to the patient’s history of antibiotic use over the preceding months [5].

In 2018, Africa Centres for Disease Control and Prevention (Africa CDC) developed a framework for antimicrobial resistance in Africa. Africa CDC is an agency of African Union (AU) that helps member states to detect, prevent, control and respond to diseases in Africa [6]. WHO declared the week of 18–24 November to be an annual antibiotic awareness campaign week with the aim of increased responsiveness of global antibiotic resistance hazard [7]. The cause of resistance to antibiotics is a topic that is receiving much attention, factors such as inappropriate use of antibiotics, bacterial gene mutations and horizontal gene transfer between bacterial species are amongst the key contributing factors. Gram-negative bacteria such as *Acinetobacter* spp., *Escherichia coli*, *Klebsiella* spp. and *Salmonella* spp. are some of the microorganisms that are extremely resistant to existing antibiotics [8]. *Escherichia coli*, *Salmonella* spp., and *Campylobacter* spp. are some of the main bacteria that cause diseases in poultry. According to the WHO antibiotics such as fluoroquinolones used in agricultural animals have resulted in the development of ciprofloxacin-resistant *Salmonella*, *Campylobacter* and *E.coli.* which contributed to human infections that were difficult to treat [2]. Apart from developing antibiotic resistance, the public can also develop an allergic reaction or liver damage on the resistance of consuming antibiotic residues in animal products [9]. *Campylobacter* spp. are prevalent in South African poultry products and pose a threat to human health [10]. It can affect the gastrointestinal tract and causes diarrheal illnesses [10]. Antibiotics that are important for treating humans must be prohibited from being used in the feed as growth-promoting [2].

There are many international programs and platforms that have been developed to address the antimicrobial resistance issue. Programs such as antibiotic stewardship, therapeutic drug committee can be used as a standard measure for collecting and comparing drug utilization patterns within and between countries [11,12]. International organizations at the forefront of addressing antimicrobial resistance such as FAO, WHO, and OIE (World Organization for Animal Health) have invested enormously on advocacy on public health risk associated with the use of antibiotics. Studies examining antimicrobial use and antibiotic resistance in Africa is widely accessible. However, coordinated surveillance and monitoring of antimicrobial resistance and use in Africa is still limited. The primary objective of this paper is to review the use of antibiotics in the poultry sector in the African continent, its impact on the public and environmental health and explore the possible alternatives available in the continent.

## 2. Antibiotic-Resistant Pathogens in Africa

Animals that are repeatedly exposed to small quantities of antibiotics can lead to the development of selective pressure amongst the microbes within the animal. The methicillin resistant *Staphylococcus aureus* ST398 has been identified in poultry and other agricultural animals and the transmission of this strain from animals to humans has been recognized [13]. Antibiotic-resistant *campylobacter* strain was discovered in the small-scale and commercial poultry farms in the province of Kwa-Zulu Natal, South Africa [14].

International meat and livestock trade are some of the factors that can contribute to the spread of resistant strains and genetic determinants [15]. Meat and egg imports from Europe to Africa could also increase the prevalence of antimicrobial strains. Between 1988 and 1989 the U.K government experienced a shock when they discovered Salmonella in eggs, this resulted in a reduction of 90% in egg consumption [16]. Furthermore, a study conducted by Papadopoulou et al. [17] reported that in Greece most pathogens that were isolated from eggs were coming from large commercial poultry farms. Where antibiotics are widely used as growth promoters and control of infectious diseases in chickens. 

Africa produces fewer antibiotics as compared to other continents [18]. Nonetheless, many antibiotics can be bought over the counter in many African countries and this practice could play a key role in worsening antibiotic resistance. Most farmers in Africa decide to purchase antimicrobial agents without consulting any animal health professional simply because they are not accessible, or farmers do not have the means to reach those [19,20]. Counterfeit medicines are an additional issue that could jeopardize the fight against antimicrobial resistance. According to Essack et al., counterfeit medicines that have reached the shores of South Africa, are mostly imported from Pakistan and India [21]. 

Namibia is one of the first countries in Africa to ban the routine use of antimicrobials in cattle [22]. The South African government has developed a strategic framework to reduce the burden of antimicrobial resistance [23]. However, it is difficult to estimate the exact prevalence of antimicrobial resistance in Africa due to the low number of antimicrobial resistance surveillance programs [22]. Antimicrobial resistance is prevalent in Africa and poses a threat to food safety and security [23]. A study that was conducted by Govender and colleagues isolated *Staphylococcus aureus* from various poultry meat products in South Africa. Twenty-one percent of the isolates selected for sensitivity testing were methicillin-resistant strains [24].

South Africa is one of the countries that are using antibiotics in food-producing animals [25]. The poultry sector in South Africa is making low-profit margin, it is also facing challenges of cheap poultry imports and has no subsidies from the government. Therefore, the immediate ban on antibiotic growth promoters in the South African poultry sector may have a short to medium term negative impact on the food security of the entire Southern African Development Community (SADC) region [26]. The immediate ban could increase the cost of the animal product because antibiotics as growth promoters help to improve feed efficiency. Using antibiotics to treat sick animals after diagnosing the disease is imperative measures that promote the welfare of animals. However, the use of antibiotics in animals feed should be regulated [2]. The development of antibiotic resistance in *Campylobacter jejuni* is likely to be influenced by using antibiotic in both healthy and sick animals in poultry farms [27]. According to a study conducted by Smith et al., the veterinary students in South Africa perceived tetracycline, penicillin and sulphonamides to be the most abused antimicrobials [28]. Bester and Essack reported that tetracycline usage in South African animals’ production system is common [29]. Moreover, tylosin has been the most extensively sold antibiotic in South Africa followed by tetracyclines, sulphonamides and penicillin [14]. Withdrawal of antibiotic before slaughtering chickens is an acceptable standard practice, however, it could be difficult to monitor if small-scale rural poultry farmers are consistently following this guideline. Another study by Fielding, isolated 102 sub-species of *K. pneumonia* in free-range chickens, which had a high level of resistance towards antibiotics ampicillin, nalidixic acid, tetracycline, and trimethoprim [30]. Similarly, a study by Bok et al., isolated *Salmonella*, *Aeromonas*, *Shigella*, *Campylobacter* and *Yersinia* retail broilers in South Africa [31]. In another report by Eagar, [32] has specified that two-third of the 1500 loads of antibiotics traded for livestock use in the 3-year period, in South Africa was mostly intended for growth promotion which included substances banned by the World Health Organization. Vancomycin is an antibiotic that is normally used to treat infections on humans. A study conducted in the Western Cape province of South Africa, showed that spent hens were 100% resistant to oxacillin, vancomycin and methicillin antibiotics, therefore, this could also pose a threat to people who eat such hens [25,33].

One health approach concept simply emphasizes that health of environment, animals and people are connected [2]. This concept could be used to manage antimicrobial-resistant and food safety concerns. Antibiotic resistance knows no boundaries and pathogens that have developed resistance to certain drugs can easily spread from countries that have good surveillance programs to those that do not, therefore, a coordinated approach is needed between developed and developing countries [34]. Resistant bacteria can be transmitted directly from human to animals and vice versa or through waste from the poultry farm to the environment. Founou et al. described antibiotics as an “endangered species” that are facing extinction because of the global development of antibiotic resistance [35]. Alternative methods that are used to reduce the use of antimicrobial-resistance include biosecurity measures, improved vaccination and good hygiene practices [36]. However, If humans have a lower good bacterium in the body as defense, they are more likely to become susceptible to resistant ones [37].

Table 1 shows the presence and prevalence of resistant *Salmonella* spp. to the antibiotic in the African continent. The table gives a summary of only 10 African states that have ad-hoc surveillance and monitoring programs of antimicrobial at their local institutions or respective farms. Most of the *Salmonella* strains were resistant to tetracycline.

## 3. Consequences of Removing Antibiotics from the Poultry Feed

The use of antibiotics is known to improve chicken health and result in weight gain. Although it comes with a cost, none the less their removal will have consequences in animal production. Cowieson and Kluenter, believe that the addition of antibiotics in animal generates substantial increases in feed conversion ratio and weight gain up to 4% [49]. This improvement must be forgone on banning the use of antibiotics or even negative growth in poor-performing flocks [49]. Moreover, the removal of antibiotic growth promoters (AGPs) could increase necrotic enteritis due to reduction in *Clostridium perfringens* control making pathogens attack more likely. A study by Cardinal et al. found that the withdrawal of antibiotics growth promoters from the diet of broilers increased the cost of production, which will eventually increase the price tag for poultry meat [50]. On the other hand, the negative effect due to the removal of prophylactic drugs may only show on the broiler performance after the first year without the use [51]. The promotion of alternative substances to antibiotics in poultry should be at the center of antimicrobials campaign [52]. Alternatives such as prebiotic and probiotic will be discussed in detail below. 

## 4. The Environmental Impact of Antibiotics

The emergence of antibiotic-resistant bacteria in the environment is a global threat to the public. The rapid spread of multiple antibiotic resistance microbes in the environment is the main concern considering the low investment in developing new antibiotics. The wastewater treatment plants are regarded as a threat to public health simply because the three-stage treatment process is insufficient to remove all the pharmaceutical residues. The wastewater treatment plants serve as carriers and transmitters of the antibiotic-resistance border between humans and the environment. Wastewater from hospitals, households and poultry farms waste contains antibiotic-resistance bacterial of animal and human origin [53]. A study conducted in the Eastern Cape province of South Africa revealed that wastewater treatment plants could be one of the contributors of sources of antibiotic-resistant *Escherichia coli* [54]. The wastewater treatment plants in West Africa are also regarded as the major sources of antibiotic-resistant bacteria. Bougnon and colleagues also reported that in Burkina Faso water from sewages used for urban agriculture may likely be one of the major sources spreading pathogens and antibacterial resistance among animals and humans [53]. 

Most rivers are considered sources of antibiotic pollution. Residues from farms and human environment may contain antibiotic-resistant genes and antibiotic substance that can contaminate the environment [53]. The emergence of antibiotic-resistant genes in the water environment is becoming a global concern. Mhlathuze River in South Africa has enteric bacteria that are resistant to antibiotic except gentamicin, the β-Lactamase gene may be widely distributed in the environment [55]. Similar findings were also reported in the Eastern Cape province of South Africa that multiple antibiotic-resistant *Pseudomonas* species were prevalent in chlorinated municipal wastewater [56]. The presence of antibiotics residues in the environment is not only the African problem. Karst river in China is widely contaminated with the presence of antibiotics. The presence of antibiotics in rivers pose a high ecological risk to the most vulnerable aquatic organisms [57]. An integrated approach could be a solution to combat antimicrobial resistance. *Pseudomonas aeruginosa* that was isolated from the environmental and clinical origin in the Benin City of Nigeria was 100% resistant to cefuroxime and amoxicillin [58].

The presence of enteric bacterial and their resistance to the antibiotic in the environment at Kakamega town in Kenya is a challenge that can cause a health hazard to the public [59]. In Kenya the highest concentration of antibiotics was found in the suburban soil of Narok town (west of Nairobi), antibiotic such as Oxytetracycline, Sulfamethoxazole, enrofloxacin and sulfamethazine were identified as the main antibiotics contaminated in soils [60]. Therefore, it is imperative that sustainable microbial monitoring program developed by the Africa CDC and WHO be implement accordingly. The information regarding the presence of antibiotic-resistant pathogens in the environment is limited in Africa. The occurrence of antibiotic-resistant bacteria in the environment is a hazard to global public health. Therefore, detailed studies with monitoring and surveillance programs could serve as a good starting point in understanding antibiotic resistance in the African environment and developing mitigation strategies thereof. 

## 5. Alternatives to Antibiotics Available in Africa

### 5.1. Probiotic + Prebiotic

Since the use of antibiotics may have a positive influence on poultry performance, excessive use as growth promoters harms human health. However, discontinuation of antibiotics affects the performance of the poultry. Some researchers argue that the use of antibiotics to improve animal performance through increased growth, and improved feed efficiency, reduces costs of meat, eggs, in other words, the consequence of banning the antibiotics will increase the cost of the animal products. It is a grim challenge which needs to be approached with a delicate balance to ensure safety and optimal performance. Researchers are looking for alternative substitutes for antibiotics in poultry production [61], such as a natural source of herbs and medicinal plants [61]. Alternatives to antibiotics, among others, include probiotics, prebiotics, competitive exclusion, enzymes and organic acids which are found to have the ability to replace antibiotics [62]. Prebiotic are potential alternatives to antibiotics used for growth promotion. They are indigestible carbohydrates source that affects the host by selectively stimulating the growth of several bacteria in the colon. The effects of prebiotics were noticed in the early 1980s as potential additives in animal feeds. The concept of prebiotic was introduced by Gibson and Robertroid in 1995 [12]. Kermanshani and Rostami reported that prebiotics will be useful replacements for antibiotics in the poultry sector due to their useful microbial population of the intestine [63]. Prebiotics have other useful functions they contribute to helping the prevention of colon cancer, minimize disease-causing bacteria like *Salmonella*, *E. coli* and ultimately change gastrointestinal microbiota [64]. 

Nutrients that are proposed to have prebiotic potential include fructans, oligofructose and inulin, fructooligosaccharides, galactan, galactooligosaccharides, resistant starch, pectin, fiber components, milk oligosaccharides [65,66]. Bacteria like bifidobacteria, lactobacilli are some of the beneficial gut microorganism that can be used as probiotic. These two species are considered the target organism for probiotic [67]. The positive effect of prebiotic in poultry is related to increasing of weight, better feed conversion ratio and low cost of therapy. Prebiotics are not supposed to be absorbed in the upper part of the gastrointestinal tract or hydrolyzed, they must be a selective substrate for a limited number of beneficial bacteria to the colon and they should stimulate their growth and activate their metabolic function and eventually, alter the colon microflora in favor of a healthier gastrointestinal environment [68]. In simple terms prebiotics are considered colonic foods, they should provide beneficial bacteria with energy, metabolic substrate and essential micronutrients.

The prebiotics helps to prevent the colonization of the digestive system with pathogens by creating unfavorable condition like altering the pH of intestinal content. Bifidobacterium, lactobacillus which is found in the digestive system has Manase enzyme. They selectively bind manna oligosaccharides only for harmful bacteria which normally do not have this enzyme [69]. The effects of mannan oligosaccharides (MOS) in broiler increases daily weight gain by 4–8% [69,70]. Kumprech et al. found that prebiotics shows similar effects like antibiotics however prebiotics has no residues and do not develop any resistance [71]. Feeding chickens MOS significantly increase the length of the villi but not the width [72]. Prebiotics have an economical and medical justification [73]. 

Probiotics are “live strains of strictly selected microorganisms which when administered in adequate amount confer a health benefit on the host” [74]. Probiotics are used in poultry feed, they have a beneficial effect on the health of the animal, stimulate growth and improve the immunity of the host [75]. The safety assessment and benefit-to risk-ratio of probiotics strains is not an easy task. Microorganisms are selected based on their health beneficial effects, they must adapt to the conditions present in the gastrointestinal tract of the given species of animals [76]. Probiotics added in feeds need to adapt to their new environment (temperature and humidity). In the European Union, the most selected probiotics are Gram-positive bacteria belonging to *Bacillus, Enterococcus, Lactobacillus, Pediococcus* and *Streptococcus*. It is not only bacteria that are used as probiotics; yeast and fungi have been used, as have strains of *Saccharomyces cerevisiae* and *Kluyveromyces*. Care should be taken as other bacteria such as enterococcus could participate in the spread of antibiotic resistance and some like bacillus cereus strains have the capacity to produce toxins [77].

The dose recommendation for most of the probiotic strains is 10^9^ colony forming units (CFU/KG) of feed. Other risk factors should be considered when mixing probiotics. The water should not contain any disinfectant or chlorine. After mixing the water should be administered within 6–12 h. Before administering probiotics, if animals were on antibiotic treatment, it is highly recommended that the treatment be withdrawn 24–48 h before administering probiotic [78]. Probiotic are natural feed additives, therefore broilers fed probiotics helps to reduce the effect of weak limbs [79]. 

Broilers that were given *Lactobacillus sporogenes* (100 mg/kg feed) increased body weight and improved feed conversion ration [80]. The addition of probiotic *lactobacilli* spp. in laying hens feed increased egg production and feed efficiency [81]. Moreover, most of the probiotics available in Africa are manufactured by developed countries and imported to Africa [82]. Notwithstanding the fact that in 2018, Deon Neveling at Stellenbosch University in South Africa developed probiotic for broilers called Gatsy [83]. In 2019, a new triple strain poultry probiotic was also launched in South Africa by another international organization [84]. Table 2 and Table 3 summarize the available prebiotic and probiotic in the poultry market, even though they are mainly manufactured in developing countries most of them can be procured online and delivered to African countries. 

### 5.2. Enzymes

Enzymes as feed additive are produced from fungi and bacteria fermentation are used for maximization of feed conversion. Enzymes such as endo-b-1-4-xylanases and b-1-3, 1-4-glucanases are commonly used with wheat and barley diets of broiler chickens for the improvement of digestibility [86]. Furthermore, the use of enzymes in poultry diets results in many benefits such as a reduction in digesta viscosity, enhanced digestion and absorption of nutrients increased feed intake and weight gain [87]. Recombinant artificial enzymes such as carbohydrases and phytases are commercially produced and traded as feed additives in monogastric food-animal production [88]. Perić et al. found that the addition of enzyme to the chicken diet resulted in high feed efficiency utilization and the final weight gain justifying the extra cost of the enzyme use [89]. However, the positive effect of enzymes is only realized when coupled with quality feed, Perić et al. realized that there was no positive effect when enzymes were added to feed a mixture of low energy and protein [90]. Khan et al. studied the influence of enzymes supplementation on the performance of laying hens and found that there was a significant increase in feed conversion ratio, egg production, egg weight and egg mass of hens [91]. Further to this, Zakaria et al. concluded that there were no monetary benefits when enzymes are included in the poultry diets [92]. In South Africa, an experiment with xylanase in the chicken diet by Mabelebele et al. recorded that, the inclusion of the enzyme had led to increasing in crude protein digestibility, feed intake and weight gain [93]. Therefore, adding enzymes to poultry diets has some mixed results.

### 5.3. Plant Extracts

Plant extracts also known as phytobiotics has been shown to be one alternative for antibiotics because of their antimicrobial, anti-inflammatory, antioxidant and antiparasitic activities, and they have been used successfully in poultry production for many years [94,95]. The other main reason behind their successful usage in poultry is because of the properties that they possess. Plant extracts have minor metabolites such as terpenoids, phenolics, glycosides, and alkaloids, present as alcohols, aldehydes, ketones, esters, ethers, and lactones [96]. These metabolites are important mechanisms which result in an increase in growth performance and health of poultry [94]. However, high usage of these secondary metabolites may result in some negative effects on digestive efficiency [97]. However, plants extracts have been reported to be safe as compared to antibiotics and they are also effective in fighting against certain bacteria [94].

In African countries plant extracts from aromatic spices (cinnamon, clove etc.), pungent spices (pepper, garlic and ginger), and herbs spices (rosemary, thyme, mint etc.). Has received increased attention over antibiotics because they are cheaper and naturally available, and they have shown to improve poultry production and health status Table 4. They are extensively used in feed as growth promoters and health protectants and their usage is also started in developed countries. The use of plant extracts in poultry production has been reported by several authors [98,99,100]. Rahimi et al. reported increase feed intake, feed conversion ratio and body weight gain improvement of endogenous digestive enzyme secretion when poultry diets were supplementation with plant extracts [101]. Whereas Al-Kassie et al. reported no adverse effects on productivity and health of broiler chickens fed plant extracts as a supplement [102]. Plants extracts have the ability to improve the gastrointestinal microbiota ecosystem by controlling pathogens volume in birds’ small intestine [103]. Moreover, Molla et al., Saminathan et al. reported that herbs like black pepper have shown to be alternative growth promoters without adversely affecting broilers performance [104,105]. Whereas other researchers reported that cineol and eucalyptol of eucalyptus and garlic extracts have the ability to prevent infectious disease, relax bird’s air sac by providing proper air circulation and improve their growth [106,107,108].

### 5.4. Organic Acids

Organic acids are also part of an effective alternative for antibiotics because of the significant role of reducing pH in the gut of poultry chickens. Reduce pH has been reported to results in improved nutrient utilization. Improved nutrient utilization is because of organic acids also being able to acidify the GIT environment which result in the activeness of protease enzyme. Moreover, they have been reported to be good at fighting against pathogenic bacteria [97]. Organic acids also decrease colonization of intestinal wall pathogens such as Salmonella and Escherichia coli which are known in damaging epithelial cells [117]. These prove that organic acids can as well used as an alternative for antibiotics to reduce pathogens load in chickens.

The most commonly known used organic acids are acetic, formic, butyric and propionic acid (simple monocarboxylic acids), malic, lactic and tartaric acids (carboxylic acids carrying a hydroxyl group on alpha carbon). In poultry, the use of these organic acids has shown to play an important role in digestion especially in diets with poor protein quality. Diet with poor protein quality results in more indigestible protein reaching GIT which end up with high protein fermentation [118]. High protein fermentation results in volatile fatty acids and ammonia and some of the unwanted gases which may cause discomfort in the animal body and adversely affect its growth rate [119]. However, the addition of organic acids has shown to improve protein and carbohydrates digestibility [120]. Several studies have been conducted on the use of organic acids as an alternative for antibiotics [121,122,123,124]. Hassan et al. reported enhanced broiler growth, feed conversion rate and nutrient utilization [121]. Whereas Chaveerach et al. reported that the addition of organic acids in drinking water for broiler chicks provided protection against Campylobacter infection [125]. Qaisrani et al. reported an increase the permeability of the bacterial cell as well as causing interference with membrane proteins and improved cell proliferation epithelial and villi height of gastrointestinal tract when ascorbic acid and citric acids were added in chickens’ drinking water [123]. Supplemented of butyric acid in broiler diets has reported improving the growth performance of chickens [122]. Table 5 summarizes the effects of organic acids on poultry performance. The organic acids such as citric acid, ascorbic acid, propionic acid and butyrate and their effects on bird performance are highlighted.

## 6. Conclusions

Both in developed and developing countries, foodborne infections continue to be an extensive and expanding public-health challenge. A report from the WHO estimated that 70% of diarrheal cases originate from bacterially infected foods. Hence, alternatives are sought to antibiotic application in food animals in order to effectively manage bacterial infections both in human and veterinary practice. Current research on alternatives to antibiotic use in food animals is slow, especially in the African continents.. Use of these drugs in humans and animals results in healthier and more prolific animal production. However, the evolution of antibiotic-resistant bacteria is probably linked with the use of these drugs such that high antibiotic utilization corresponds to greater and faster emergence of antimicrobial-resistant bacterial strains. Finally, improved sanitary farm conditions, as well as the maintenance of farm biosecurity, are important alternatives that could be adopted by farmers instead of depending on antibiotic drugs for disease control and prevention. These would essentially serve as a means of preventing the entry and dissemination of disease amongst animals. The available alternatives (probiotics, prebiotics, enzymes, plant extract and organic acids) to antibiotics have a potential role in lowering dependency on the existing antimicrobial substances. Failure to implement the WHO antimicrobial usage recommendations could deteriorate the situation and intensify the burden of diseases or increase mortality rate in Africa and other continents. Therefore, CDC Africa could assist in ensuring that its member states adopt the available alternatives to antibiotics.

## Figures and Tables

**Table 1:** Presence of varying concentration of Salmonella and antibiotic resistance in Poultry farms in 10 African countries.

Country	Antibiotic Resistance	Concentration	Species/Sample	Sources
Nigeria	Oxacillin (100%) Ampicillin (96%), Tylosin (93.9%), Ceftazidime (83.7%) Oxytetracycline (63.3%)	Six *Salmonella* isolates were identified: *S. Gallinarum* 57.2% *S. Typhimurium* 8.2%, *S. Typhi* 20.4%, *S. Pullorum* 6.1%, *S. Enteritidis* 6.1% *S. Paratyphi* A 2.0%.	Poultry droppings poultry feeds, feces and hand swabs from poultry farm workers.	Agada et al. [38]
Mauritius	100% resistance to Tetracycline, Erythromycin (80%), Streptomycin (80%), Chloramphenicol (60%)	17% were found to be positive for *Salmonella*,	Seven Samples of poultry intestine, litter of two different farms (7) and eggs (9)	Phagoo and Neetoo [39]
Zambia	2 of the 5 *Salmonella* isolates were resistant to at least 1 antibiotic.	Five *Salmonella* isolates were identified.	Samples collected from broiler chickens obtained from local markets and shops	Muonga et al. [40]
Burkino Faso	Resistance to ampicillin, chloramphenicol, streptomycin, sulfonamides and trimethoprim was detected	55% of the poultry samples tested positive	350 samples (Poultry feces)	Kagambega et al. [41]
South Africa	*Salmonella* isolates showed resistance to nearly all ten antimicrobial agents used.	*InvA* gene was used to test for *Salmonella* and 51% of samples tested positive	200 chicken samples	Zishir et al. [42]
Chad	*S. Limete* resistant to 3 antibiotics and *S. Minnesota* isolates resistant to 5 diverse antimicrobial classes	*Salmonella* isolates identified were: *Salmonella* Colindale (19%) S. Minnesota (18%). Below 10% were S. Havana and S. Riggil, S. Kottbus and S. Amager, S. Idikan, Mississipi, and Muenchen	laying hens and broiler chicken	Tabo et al. [43]
Ethiopia	30 isolates were resistant to one or more of antibiotics. Of 30, 19 were multidrug resistant while 11 isolates resistant to tetracycline. One isolate was resistant Kanamycin. Other isolates were tetra-, penta-, hexa-, and hepta resistant, correspondingly. 31 isolates susceptible to Gentamycin and Ciprofloxacin	Out of 205 samples collected, 31 (15.12%) isolates were detected.	In total, 205 samples were collected, namely: 100 cloacal swabs, 75 fresh feces, 10 litter samples, 8 chicken feed samples, 8 poultry drinking water and 4 farmworker hand swab samples	Abunna et al. [44]
Ghana	Resistance: Nalidixic acid (89.5%), tetracycline (80·7%), ciprofloxacin (64.9%), sulfamethazole (42·1%), trimethoprim (29.8%) and ampicillin (26·3%).All strains were susceptible to cefotaxime, ceftazidime and cefoxitin.	Out of 200 samples collected, *Salmonella* was detected in 94 samples (47%)	egg-laying hens and broilers. Sampling of feces (75), dust (75), feed (10) and drinking water (10) was performed at 75 poultry farms in Ghana and skin neck (30) at a local abattoir	Andoh et al. [45]
Senegal	Resistance: trimethoprim-sulfamethoxazole, tetracycline, trimethoprim, streptomycin, and sulfonamides. All Salmonella serovars were susceptible to fluoroquinolones and cephalosporins	*Salmonella* was detected in chicken fa\eces (35.1%), on carcass skin (38.6%), and in muscle (29.8%) of farms, respectively. *Salmonella* detected in chicken meat servings from 14.3% of the street restaurants and in 40.4% of the chicken carcasses tested	Chicken feces, carcass skin and muscle	Dione et al. [46]
Morocco	Resistance to tetracycline, sulfamides, trimethoprim and streptomycin was detected.	57 were positive for *Salmonella*, 30 out of 57 from local market, 24 out of 57 from artisanal slaughterhouses and 3 out of 57 from poulterers’ shops	576 samples were collected: local market (144), artisanal slaughterhouses (144), poultry shops (144) and from a supermarket (144)	Abdellah et al. [47]
Botswana	All samples were resistant to tetracycline, Ampicillin and Sulphatriad but susceptible to Gentamycin	Chicken livers had 50% salmonella, intestine 29%; Ostrich small intestine 16% liver 12.9%	128 chicken samples; 124 Ostrich. 32 livers, gall bladder, small intestine and large intestine. For Ostrich 31 livers, small intestine, large intestine and cloacae	Gaedirelwe and Sebunya [48]

**Table 2:** Examples of prebiotics used in poultry [85].

Prebiotic Substance	Trade Name
Polysaccharides, Oligosaccharides	Bacto CS 1000
MOS, β-Glucans	DOISORB DN (Dolfos)
MOS, β-Glucans	Metsac MOS (Vitjira)
β-Glucans	Mycocyd forte (Herbline)
MOS, β-Glucans	Mycostop (Extra-vit)
ScFOS (Short chain Fructo-oligosaccharides)	Profeed (Beghnir Meiji)

**Table 3:** Examples of probiotic used in poultry [85].

Microorganism	Trade Name
*Bacillus amyloliquefaciens*	Ecobiol (Norel Animal nutrition)
*Bacillus subtilis*	Calsporin (ORFFA); Enviva pro (Danisco Animal nutrition); Gallipro (Evonik industries)
*Bifidobacterium Bifidum, Lactobacillus acidophilus; Pediococcus Faecium*	Blogen D (Bio-gen)
*Enterococcus faecium*	B.I.O. sol (Biochem); Gallvit Probiotyk (Galvit)
*Lactobacillus acidophilus, casei, plantarum*	Cerbiogalli
*Lactobacillus: Rhamnosus, Farciminis*	Ecobiol (Novel Animal nutrition)
*Lactobacillus Salivarius, Pediococcus parvilus*	Floramax-B11 (Pacific vet group)

**Table 4:** Plants extracts, active compounds and their functions and effect on poultry.

Plant Extract	Active Compound	General Function	Effect in Poultry	Sources
Aromatic spices				
Cinnamon	Cinnamaldehyde	Appetite and digestion stimulant, antiseptic	Improved feed efficiency and body weight an increase in carcass energy retention and an increase in carcass protein retention.	Al-Kassie [109]; Akyildiz and Denli [94] Cross et al. [110]
Cloves	Eugenol	Appetite and digestion stimulant, antiseptic		Akyildiz and Denli [94]; Chisoro [111]
Pungent spices				
Pepper	Piperine	Digestion stimulant	No effect on live performance or in organ morphometrics	Barreto et al. [112]; Akyildiz and Denli [94]
Garlic	Allicin	Digestion stimulant, antiseptic	Higher body weights	Akyildiz and Denli [94]; Chisoro [111]
Ginger	Zingerone	Gastric stimulant	No effects on performance	Mohammed and Yusuf [113]; Akyildiz and Denli [94]
Herbs spices				
Rosemary	Cineol	Digestion stimulant, antiseptic, antioxidant	Improved live weight and Feed efficiency	Yesilbag et al. [114]; Mathlouthi et al. [115]; Akyildiz and Denli [94]; Cross et al. [110]
Thyme	Thymol	Digestion stimulant, antiseptic, antioxidant	No significant effect on BW/FCR Improve BW and FCR; No effect on the intestinal microflora populations	Pourmahmoud et al. [116]; Al-Kassie [99]; Akyildiz and Denli [94]; Cross et al. [110]
Mint	Menthol	Appetite and digestion stimulant, antiseptic	The best result for production percentage, feed conversion ratio, shell thickness and yolk weight in layers. Reduction in serum total cholesterol, triglycerides and low-density lipoprotein (LDL) concentration	Akyildiz and Denli [94]; Chisoro [111]

**Table 5:** Effects of organic acids on poultry performance.

Organic Acids	Findings	Sources
Citric acid	Improvement in ileal nutrient digestibility, cell proliferation epithelial and villi height	Nourmohammadi et al. [126] Qaisrani et al. [122]; Mohammadagheri et al., [124]
Ascorbic acid	Improved cell proliferation epithelial and villi height	Qaisrani et al. [122]; Denli and Demirel [127]
Propionic acid and sodium bentonite	Increase in digestibility and availability of nutrients (such as calcium and total phosphorus	Ziaie et al. [128]; Denli and Demirel [127]
Butyrate	Increased body weight, improved feed efficiency	Panda et al. [129]; Denli and Demirel [127]

## References

[1] World Health Organization (2003). Joint FAO/OIE/WHO Expert Workshop on Non-Human Antimicrobial Usage and Antimicrobial Resistance: Scientific Assessment.

[2] World Health Organization (2017). WHO Guidelines on Use of Medically Important Antimicrobials in Food-Producing Animals: Web Annex A: Evidence Base.

[3] O’Neill J. (2016). Review on Antimicrobial Resistance: Tackling Drug-Resistant Infections Globally: Final Report and Recommendations. https://amr-review.org/sites/default/files/160518_Final%20paper_with%20cover.pdf.

[4] Shallcross L.J., Davies S.C. (2014). The World Health Assembly resolution on antimicrobial resistance. J. Antimicrob. Chemother..

[5] Alkindi F.F., Yulia R., Herawati F., Jaelani A.K. (2019). Influence of historical use of antibiotics toward antibiotic resistance. Farmasains J. Farm. dan Ilmu Kesehat..

[6] Varma J.K., Oppong-Otoo J., Ondoa P., Perovic O., Park B.J., Laxminarayan R., Peeling R.W., Schultsz C., Li H., Ihekweazu C. (2018). Africa Centres for Disease Control and Prevention’s framework for antimicrobial resistance control in Africa. Afr. J. Lab. Med..

[7] Canon A. (2019). The American Association of Swine Veterinarians Antibiotic Awareness Week and AASV’s Commitment to the AMR Challenge. https://www.aasv.org/shap/issues/v27n6/v27n6advocacy.html.

[8] Carlet J., Jarlier V., Harbarth S., Voss A., Goossens H., Pittet D. (2012). Ready for a world without antibiotics? The Pensières Antibiotic Resistance Call to Action. Antimicrob. Resist. Infect. Control..

[9] World Health Organization (2011). Tackling Antibiotic Resistance from a Food Safety Perspective in Europe.

[10] Bartkowiak-Higgo A.J., Veary C.M., Venter E.H., Bosman A.M. (2006). A pilot study on post-evisceration contamination of broiler carcasses and ready-to-sell livers and intestines (mala) with Campylobacter jejuni and Campylobacter coli in a high-throughput South African poultry abattoir. J. S. Afr. Veter. Assoc..

[11] Massele A., Tiroyakgosi C., Matome M., Desta A., Müller A., Paramadhas B.D.A., Malone B., Kurusa G., Didimalang T., Moyo M. (2016). Research activities to improve the utilization of antibiotics in Africa. Expert Rev. Pharmacoecon. Outcomes Res..

[12] Gibson G.R., Roberfroid M.B. (1995). Dietary Modulation of the Human Colonic Microbiota: Introducing the Concept of Prebiotics. J. Nutr..

[13] Pantosti A. (2012). Methicillin-Resistant Staphylococcus aureus Associated with Animals and Its Relevance to Human Health. Front. Microbiol..

[14] Bester L.A., Essack S.Y. (2012). Observational Study of the Prevalence and Antibiotic Resistance of Campylobacter spp. from Different Poultry Production Systems in KwaZulu-Natal, South Africa. J. Food Prot..

[15] Buhr B.L. (2003). Traceability and information technology in the meat supply chain: Implications for firm organization and market structure. J. Food Distrib. Res..

[16] Oboegbulem S., Collier P., Sharp J., Reilly W. (1993). Epidemiological aspects of outbreak of food-borne salmonellosis in Scotland between 1980 and 1989. Rev. Sci. Tech..

[17] Papadopoulou C., Dimitriou D., Levidiotou S., Gessouli H., Panagiou A., Golegou S., Antoniades G. (1997). Bacterial strains isolated from eggs and their resistance to currently used antibiotics: Is there a health hazard for consumers?. Comp. Immunol. Microbiol. Infect. Dis..

[18] Gelband H., Molly Miller P., Pant S., Gandra S., Levinson J., Barter D., White A., Laxminarayan R. (2015). The state of the world’s antibiotics 2015. Wound Health S. Afr..

[19] Alonso C.A., Zarazaga M., Ben Sallem R., Jouini A., Ben Slama K., Torres C. (2017). Antibiotic resistance in Escherichia coli in husbandry animals: The African perspective. Lett. Appl. Microbiol..

[20] Ndihokubwayo J.B., Yahaya A.A., Desta A.T., Ki-Zerbo G., Odei E.A., Keita B., Pana A.P., Nkhoma W. (2013). Antimicrobial resistance in the African Region: Issues, challenges and actions proposed. Afr. Health Monit..

[21] Essack S.Y., Schellack N., Pople T., Van Der Merwe L., Suleman F., Meyer J.C., Gous A.G.S., Benjamin D. (2011). Part III. Antibiotic supply chain and management in human health. S. Afr. Med. J..

[22] Medina M.J., Legido-Quigley H., Hsu L.Y. (2020). Antimicrobial Resistance in One Health. Global Health Security.

[23] Founou L.L., Amoako D.G., Founou R.C., Essack S.Y. (2018). Antibiotic Resistance in Food Animals in Africa: A Systematic Review and Meta-Analysis. Microb. Drug Resist..

[24] Govender V., Madoroba E., Magwedere K., Fosgate G.T., Kuonza L. (2019). Prevalence and risk factors contributing to antibiotic-resistant Staphylococcus aureus isolates from poultry meat products in South Africa, 2015–2016. J. S. Afr. Veter. Assoc..

[25] Moyane J.N., Jideani A.I.O., Aiyegoro O.A. (2013). Antibiotics usage in food-producing animals in South Africa and impact on human: Antibiotic resistance. Afr. J. Microbiol. Res..

[26] Theobald S., Etter E.M., Gerber D., Abolnik C. (2019). Antimicrobial Resistance Trends in Escherichia coli in South African Poultry: 2009–2015. Foodborne Pathog. Dis..

[27] Van T.T.H., Yidana Z., Smooker P.M., Coloe P.J. (2020). Antibiotic use in food animals worldwide, with a focus on Africa: Pluses and minuses. J. Glob. Antimicrob. Resist..

[28] Smith P.W., Agbaje M., LeRoux-Pullen L., Van Dyk D., Debusho L.K., Shittu A., Sirdar M., Fasanmi O., Adebowale O.O., Fasina F. (2019). Implication of the knowledge and perceptions of veterinary students of antimicrobial resistance for future prescription of antimicrobials in animal health, South Africa. J. S. Afr. Veter Assoc..

[29] Bester L.A., Essack S.Y. (2008). Prevalence of antibiotic resistance in Campylobacter isolates from commercial poultry suppliers in KwaZulu-Natal, South Africa. J. Antimicrob. Chemother..

[30] Fielding B.C., Mnabisa A., Gouws P.A., Morris T. (2012). Antimicrobial-resistant Klebsiella species isolated from free-range chicken samples in an informal settlement. Arch. Med. Sci..

[31] Bok H.E., Holzapfel W.H., Odendaal E.S., Van Der Linde H.J. (1986). Incidence of foodborne pathogens on retail broilers. Int. J. Food Microbiol..

[32] Eagar H., Swan G., Van Vuuren M. (2012). A survey of antimicrobial usage in animals in South Africa with specific reference to food animals. J. S. Afr. Veter. Assoc..

[33] Garcés L. (2002). The Detrimental Impacts of Industrial Animal Agriculture: A Case for Humane and Sustainable Agriculture, Compassion in World Farming Trust. http://www.ciwf.org.uk/includes/documents/cm_docs/2008/d/detrimental_impact_industrial_animal_agriculture_2002.pdf.

[34] World Health Organization (2017). Global Antimicrobial Resistance Surveillance System (GLASS) Report: Early Implementation 2016–2017.

[35] Founou L.L., Founou R.C., Essack S.Y. (2016). Antibiotic Resistance in the Food Chain: A Developing Country-Perspective. Front. Microbiol..

[36] Magnusson U., Sternberg S., Eklund G., Rozstalnyy A. (2019). Prudent and Efficient Use of Antimicrobials in Pigs and Poultry.

[37] Schloss P.D. (2014). Microbiology: An integrated view of the skin microbiome. Nature.

[38] Agada G.O.A., Abdullahi I., Aminu M., Odugbo M., Chollom S.C., Kumbish P.R., Okwori A.E.J. (2014). Prevalence and Antibiotic Resistance Profile of Salmonella Isolates from Commercial Poultry and Poultry Farm-handlers in Jos, Plateau State, Nigeria. Br. Microbiol. Res. J..

[39] Phagoo L., Neetoo H. (2015). Antibiotic resistance of Salmonella in poultry farms of Mauritius. J. Worlds Poult. Res..

[40] Muonga E.M., Mainda G., Mukuma M., Kwenda G., Hang’ombe B., Phiri N., Mwansa M., Munyeme M., Muma J.B. Antimicrobial Resistance of Escherichia Coli and Salmonella Isolated from Raw Retail Broiler Chickens in Zambia. https://www.researchsquare.com/article/rs-44168/v1.

[41] Kagambèga A., Lienemann T., Aulu L., Traore A.S., Barro N., Siitonen A., Haukka K. (2013). Prevalence and characterization of Salmonella enterica from the feces of cattle, poultry, swine and hedgehogs in Burkina Faso and their comparison to human Salmonella isolates. BMC Microbiol..

[42] Zishiri O.T., Mkhize N., Mukaratirwa S. (2016). Prevalence of virulence and antimicrobial resistance genes in Salmonella spp. isolated from commercial chickens and human clinical isolates from South Africa and Brazil. Onderstepoort J. Veter. Res..

[43] Tabo D.-A., Diguimbaye C.D., Granier S.A., Moury F., Brisabois A., Elgroud R., Millemann Y. (2013). Prevalence and antimicrobial resistance of non-typhoidal Salmonella serotypes isolated from laying hens and broiler chicken farms in N’Djamena, Chad. Veter. Microbiol..

[44] Abunna F., Bedasa M., Beyene T., Ayana D., Mamo B., Duguma R. (2016). Salmonella: Isolation and antimicrobial susceptibility tests on isolates collected from poultry farms in and around Modjo, Central Oromia, and Ethiopia. JAPSC.

[45] Andoh L.A., Dalsgaard A., Obiri-Danso K., Newman M.J., Barco L., Olsen J.E. (2016). Prevalence and antimicrobial resistance ofSalmonellaserovars isolated from poultry in Ghana. Epidemiology Infect..

[46] Dione M.M., Ieven M., Garin B., Marcotty T., Geerts S. (2009). Prevalence and Antimicrobial Resistance of Salmonella Isolated from Broiler Farms, Chicken Carcasses, and Street-Vended Restaurants in Casamance, Senegal. J. Food Prot..

[47] Abdellah C., Fouzia R.F., Abdelkader C., Rachida S.B., Mouloud Z. (2009). Prevalence and anti-microbial susceptibility of Salmonella isolates from chicken carcasses and giblets in Mekns, Morocco. Afr. J. Microbiol. Res..

[48] Gaedirelwe O.G., Sebunya T.K. (2008). The Prevalence and Antibiotic Susceptibility of Salmonella sp. Poultry and ostrich Samples from Slaughter Houses in Gaborone, Botswana. J. Anim. Vet. Adv..

[49] Cowieson A.J., Kluenter A. (2019). Contribution of exogenous enzymes to potentiate the removal of antibiotic growth promoters in poultry production. Anim. Feed. Sci. Technol..

[50] Cardinal K., Kipper M., Andretta I., Ribeiro A.M.L. (2019). Withdrawal of antibiotic growth promoters from broiler diets: Performance indexes and economic impact. Poult. Sci..

[51] Engster H.M., Marvil D., Stewart-Brown B. (2002). The Effect of Withdrawing Growth Promoting Antibiotics from Broiler Chickens: A Long-Term Commercial Industry Study. J. Appl. Poult. Res..

[52] Dela Cruz P.J.D., Dagaas C.T., Mangubat K.M.M., Angeles A.A., Abanto O.D. (2019). Dietary effects of commercial probiotics on growth performance, digestibility, and intestinal morphometry of broiler chickens. Trop. Anim. Health Prod..

[53] Bougnom B.P., Zongo C., McNally A., Ricci V., Etoa F.X., Thiele-Bruhn S., Piddock L.J.V. (2019). Wastewater used for urban agriculture in West Africa as a reservoir for antibacterial resistance dissemination. Environ. Res..

[54] Igwaran A., Iweriebor B.C., Okoh A.I. (2018). Molecular Characterization and Antimicrobial Resistance Pattern of Escherichia coli Recovered from Wastewater Treatment Plants in Eastern Cape South Africa. Int. J. Environ. Res. Public Health.

[55] Lin J., Biyela P., Puckree T. (2004). Antibiotic resistance profiles of environmental isolates from Mhlathuze River, KwaZulu-Natal (RSA). Water SA.

[56] Odjadjare E.E., Igbinosa E.O., Mordi R., Igere B.E., Igeleke C.L., Okoh A.I. (2012). Prevalence of Multiple Antibiotics Resistant (MAR) Pseudomonas Species in the Final Effluents of Three Municipal Wastewater Treatment Facilities in South Africa. Int. J. Environ. Res. Public Heal..

[57] Xue B., Zhang R., Wang Y., Liu X., Li J., Zhang G. (2013). Antibiotic contamination in a typical developing city in south China: Occurrence and ecological risks in the Yongjiang River impacted by tributary discharge and anthropogenic activities. Ecotoxicol. Environ. Saf..

[58] Isichei-Ukah O., Enabulele O. (2018). Prevalence and antimicrobial resistance of Pseudomonas aeruginosa recovered from environmental and clinical sources in Benin City, Nigeria. Ife J. Sci..

[59] Malaho C., Wawire S.A., Shivoga W.A. (2018). Antimicrobial Resistance Patterns of Enterobacteriaceae Recovered from Wastewater, Sludge and Dumpsite Environments in Kakamega Town, Kenya. Afr. J. Microbiol. Res..

[60] Yang Y., Owino A.A., Gao Y., Yan X., Xu C., Wang J. (2016). Occurrence, composition and risk assessment of antibiotics in soils from Kenya, Africa. Ecotoxicology.

[61] Kermanshahi H., Rostami H. (2006). Influence of supplemental dried whey on broiler performance and cecal lora. Int. J. Poult. Sci..

[62] Cummings J.H., Macfarlane G. (2002). Gastrointestinal effects of prebiotics. Br. J. Nutr..

[63] Khan R.U., Naz S., Nikousefat Z., Tufarelli V., Laudadio V. (2012). Thymus vulgaris: Alternative to antibiotics in poultry feed. World’s Poult. Sci. J..

[64] Doyle M.E. (2001). Alternatives to Antibiotic Use for Growth Promotion in Animal Husbandry.

[65] Bird A.R., Conlon M.A., Christophersen C.T., Topping D.L. (2010). Resistant starch, large bowel fermentation and a broader perspective of prebiotics and probiotics. Benef. Microbes.

[66] Coppa G.V., Zampini L., Galeazzi T., Gabrielli O. (2006). Prebiotics in human milk: A review. Dig. Liver Dis..

[67] Rastall R.A., Gibson G.R. (2015). Recent developments in prebiotics to selectively impact beneficial microbes and promote intestinal health. Curr. Opin. Biotechnol..

[68] Gibson G.R., Fuller R. (2000). Aspects of in vitro and in vivo research approaches directed toward identifying probiotics and prebiotics for human use. J. Nutr..

[69] Sinovec Z., Markovic R. (2005). Use of pre-biotics in poultry nutrition. Biotehnol. Stoc..

[70] Shashidhara R.G., Devegowda G. (2003). Effect of dietary mannan oligosaccharide on broiler breeder production traits and immunity. Poult. Sci..

[71] Kumprecht I., Zobac P. (1998). The effect of probiotic preparations containing *Saccharomyces cerevisae* and *Enterococcus faecium* in diets with different levels of beta-vitamins on chicken broiler performance. Czech J. Anim. Sci. UZPI.

[72] Petersen C.B., Lyons T.P. (1998). Comparative effects of ZooLac, Bio-MOS and Bio-Pro on performance of broilers to 36 days. Poster. Biotechnology in the Feed Industry. Proc. Alltechs 14th Annual Symposium.

[73] Spring P. (1996). Effects of Mannanoligosaccharide on Different Cecal Parameters and on Cecal Concentrations of enteric Pathogens in Poultry. Ph.D. Thesis.

[74] Indikova I., Humphrey T.J., Hilbert F. (2015). Survival with a Helping Hand: Campylobacter and Microbiota. Front. Microbiol..

[75] Guyard-Nicodème M., Keita A., Quesne S., Amelot M., Poëzévara T., Le Berre B., Sánchez J., Vesseur P., Martin A., Medel P. (2016). Efficacy of feed additives against Campylobacter in live broilers during the entire rearing period. Poult. Sci..

[76] Isolauri E., Salminen S., Ouwehand A.C. (2004). Probiotics. Best Pr. Res. Clin. Gastroenterol..

[77] Patel S.G., Raval A.P., Bhagwat S.R., Sadrasaniya D.A., Patel A.P., Joshi S.S. (2015). Effects of Probiotics Supplementation on Growth Performance, Feed Conversion Ratio and Economics of Broilers. J. Anim. Res..

[78] Mizak L., Gryko R., Kwiatek M., Parasion S. (2012). Probiotics in animal nutrition. Życie Weter..

[79] Anadón A., Martínez-Larrañaga M.R., Martínez M. (2006). Probiotics for animal nutrition in the European Union. Regulation and safety assessment. Regul. Toxicol. Pharmacol..

[80] Plavnik I., Scott M.L. (1980). Effects of Additional Vitamins, Minerals, or Brewer’s Yeast upon Leg Weaknesses in Broiler Chickens. Poult. Sci..

[81] Panda A.K., Reddy M., Rao S.R., Praharaj N. (2003). Production performance, serum/yolk cholesterol and immune competence of white leghorn layers as influenced by dietary supplementation with probiotic. Trop. Anim. Health Prod..

[82] Sekhon B.S., Jairath S. (2010). Prebiotics, probiotics and synbiotics: An overview. J. Pharm. Educ. Res..

[83] Deon N. Stellenbosch University Student Develops First Gut Probiotic for Broiler Chickens. https://www.bizcommunity.com/Article/196/815/185300.html.

[84] Hansen C. Internationally Renowned Chr. Hansen Launched Unique Triple Strain Probiotic for Poultry in South Africa. www.chr-hansen.com.

[85] Markowiak P., Slizewska K. (2018). The role of probiotics, prebiotics and synbiotics in animal nutrition. Gut Pathog..

[86] Cowieson A.J., Hruby M., Pierson E.E.M. (2006). Evolving enzyme technology: Impact on commercial poultry nutrition. Nutr. Res. Rev..

[87] Khattak F.M., Pasha T.N., Hayat Z., Mahmud A. (2006). Enzyme in poultry nutrition. J. Anim. Plant Sci..

[88] Adeola O., Cowieson A.J. (2011). Board-invited review: Opportunities and challenges in using exogenous enzymes to improve non-ruminant animal production. J. Anim. Sci..

[89] Perić L., Milošević N., Djukic-Stojcic M., Bjedov S., Rodic V. (2008). Effect of enzymes on performances of broiler chickens. Biotehnol. Stoc..

[90] Perić L., Kovčin S., Stanaćev V., Milošević N. (2002). Effect of enzymes on broiler chick performance. Bul. USAMV.

[91] Khan S.H., Atif M., Mukhtar N., Rehman A., Fareed G. (2011). Effects of supplementation of multi-enzyme and multi-species probiotic on production performance, egg quality, cholesterol level and immune system in laying hens. J. Appl. Anim. Res..

[92] Alal M.A., Zakaria H.A., Jabarin A.S. (2008). Effect of Exogenous Enzymes on the Growing Performance of Broiler Chickens Fed Regular Corn/Soybean-Based Diets and the Economics of Enzyme Supplementation. Pak. J. Nutr..

[93] Mabelebele M., Gous R.M., Siwela M., O’Neil H., Iji P. (2017). Performance of broiler chickens fed South African sorghum-based diets with xylanase. S. Afr. J. Anim. Sci..

[94] Akyildiz S., Denli M. (2016). Application of plant extracts as feed additives in poultry nutrition. Anim. Sci..

[95] Karangiya V.K., Savsani H.H., Patil S.S., Garg D., Murthy K.S., Ribadiya N.K., Vekariya S.J. (2016). Effect of dietary supplementation of garlic, ginger and their combination on feed intake, growth performance and economics in commercial broilers. Veter. World.

[96] Cao G.T., Zeng X.F., Chen A.G., Zhou L., Zhang L., Xiao Y.P. (2013). Effects of a probiotic, Enterococcus faecium, on growth performance, intestinal morphology, immune response, and caecal microflora in broiler chickens challenged with Escherichia coli. Poult. Sci..

[97] Yadav A.S., Kolluri G., Gopi M., Karthik K., Malik Y.S., Dhama K. (2016). Exploring alternatives to antibiotics as health promoting agents in poultry- a review. J. Exp. Boil. Agric. Sci..

[98] Elagib H.A.A., Elamin W.I.A., Elamin K.M., Malik H.E.E. (2013). Effect of Dietary Garlic (Allium sativum) Supplementation as Feed Additive on Broiler Performance and Blood Profile. J. Anim. Sci. Adv..

[99] Gopi M., Purushothaman M.R., Chandrasekaran D. (2014). Effect of dietary coenzyme Q10 supplementation on the growth rate, carcass characters and cost effectiveness of broiler fed with three energy levels. SpringerPlus.

[100] Heinzl I., Borchardt T. (2015). Secondary plant compound to reduce the use of antibiotics?. Int. Poult. Prod..

[101] Rahimi S., Teymouri Z.Z., Karimi T.M., Omidbaigi R., Rokni H. (2011). Effect of the three herbal extracts on growth performance, immune system, blood factors and intestinal selected bacterial population in broiler chickens. J. Agric. Sci. Technol..

[102] Al-Kassie G.A., Mamdooh A.M.A., Saba J.A. (2011). The effects of using hot red pepper as a diet supplement on some performance traits in broiler. Pak. J. Nutr..

[103] Hashemi S.R., Davoodi H. (2011). Herbal plants and their derivatives as growth and health promoters in animal nutrition. Veter. Res. Commun..

[104] Molla, Rahman M., Akter F., Mostofa M. (2013). Effects of Nishyinda, black pepper and cinnamon extract as growth promoter in broilers. Bangladesh Veter..

[105] Saminathan M., Rai R.B., Dhama K., Tiwari R., Chakraborty S., Amarpal R.G.J., Kannan K. (2013). Systematic review on anticancer potential and other health beneficial pharmacological activities of novel medicinal plant *Morindacitri folia* (Noni). Int. J. Pharmacol..

[106] Dhama K., Tiwari R., Chakrabort S., Saminathan M., Kumar A., Karthik K., Wani M.Y., Singh S., Rahal A. (2014). Evidence Based Antibacterial Potentials of Medicinal Plants and Herbs Countering Bacterial Pathogens Especially in the Era of Emerging Drug Resistance: An Integrated Update. Int. J. Pharmacol..

[107] Dhama K., Karthik K., Tiwari R., Shabbir M.Z., Barbuddhe S., Malik S.V.S., Singh R.K. (2015). Listeriosis in animals, its public health significance (food-borne zoonosis) and advances in diagnosis and control: A comprehensive review. Veter. Q..

[108] Nakielski A. (2015). Treating respiratory tract infections in poultry with the use of herbs. Int. Poult. Pract..

[109] Al-Kassie G.A. (2009). Influence of two plant extracts derived from thyme and cinnamon on broiler performance. Pak. Vet. J..

[110] Cross D.E., McDevitt R.M., Hillman K., Acamovic T. (2007). The effect of herbs and their associated essential oils on performance, dietary digestibility and gut microflora in chickens’ from7 to 28 days of age. Britian Poult. Sci..

[111] Chisoro P. (2016). Plant Extracts as Alternatives to Antibiotics in Animal Feed.

[112] Barreto M., Menten J., Racanicci A., Pereira P., Rizzo P. (2008). Plant extracts used as growth promoters in broilers. Revista Brasileira de Ciência Avícola.

[113] Mohammed A.A., Yusuf M. (2011). Evaluation of ginger (*Zingiber officinale*) as a feed additive in broiler diets. Livest. Res. Rural. Dev..

[114] Yesilbag D., Eren M., Agel H., Kovanlikaya A., Balci F. (2011). Effects of dietary rosemary, rosemary volatile oil and vitamin Eon broiler performance meat quality and serum SOD activity. Br. Poult. Sci..

[115] Mathlouthi N., Bouzaienne T., Oueslati I., Recoquillay F., Hamdi M., Urdaci M., Bergaoui R. (2012). Use of rosemary, oregano, and a commercial blend of essential oils in broiler chickens invitro antimicrobial activities and effects on growth performance. J. Anim. Sci..

[116] Pourmahmoud B., Aghazadeh A., Sis N.M. (2013). The Effect of Thyme Extract on Growth Performance, Digestive Organ Weights and Serum Lipoproteins of Broilers Fed Wheat-Based Diets. Ital. J. Anim. Sci..

[117] Hermans D., De Laet M. (2014). Reaching genetic potential with medium chain fatty acids (MCFAs). Int. Poult. Prod..

[118] Diether N.E., Willing B.P. (2019). Microbial fermentation of dietary protein: An important factor in diet⁻Microbe⁻Host Interaction. Microorganisms.

[119] Ikker P., Dirkzwager A., Fledderus J., Trevisi P., Le Huërou-Luron I., Lallès J., Awati A. (2007). Dietary protein and fermentable carbohydrates contents influence growth performance and intestinal characteristics in newly weaned pigs. Livest. Sci..

[120] Adil S., Banday M.T., Bhat G.A., Mir M.S., Rehman M. (2010). Effect of Dietary Supplementation of Organic Acids on Performance, Intestinal Histomorphology, and Serum Biochemistry of Broiler Chicken. Veter. Med. Int..

[121] Hassan H.M.A., Mohamed M.A., Youssef A.W., Hassan E.R. (2010). Effect of Using Organic Acids to Substitute Antibiotic Growth Promoters on Performance and Intestinal Microflora of Broilers. Asian Australas. J. Anim. Sci..

[122] Qaisrani S., Van Krimpen M., Kwakkel R., Verstegen M., Hendriks W. (2015). Diet structure, butyric acid, and fermentable carbohydrates influence growth performance, gut morphology, and cecal fermentation characteristics in broilers. Poult. Sci..

[123] Abdurrahman Z.H., Pramono Y.B., Suthama N. (2016). Meat Characteristic of Crossbred Local Chicken Fed Inulin of Dahlia Tuber and Lactobacillus sp.. Media Peternak..

[124] Mohammadagheri N., Najafi R., Najafi R. (2016). Effects of dietary supplementation of organic acids and phytase on performance and intestinal histomorphology of broilers. Veter. Res. Forum Int. Q. J..

[125] Chaveerach P., Keuzenkamp D.A., Lipman L.J.A., Van Knapen F. (2004). Effect of Organic Acids in Drinking Water for Young Broilers on Campylobacter Infection, Volatile Fatty Acid Production, Gut Microflora and Histological Cell Changes. Poult. Sci..

[126] Nourmohammadi R., Hosseini S.M., Farhangfar H., Bashtani M. (2012). Effect of citric acid and microbial phytase enzyme on ileal digestibility of some nutrients in broiler chicks fed corn-soybean meal diets. Ital. J. Anim. Sci..

[127] Denli M. (2018). Replacement of antibiotics in poultry diets. CAB Rev..

[128] Ziaie H., Bashtani M., Karimi T.M.A., NaeeimiIpour H., Farhangfar H., Zeinai A. (2011). Effect of antibiotic and its alternatives on morphometric characteristics, mineral content and bone strength of tibia in Ross broiler chickens. Glob. Vet..

[129] Panda A.K., Rao S.V.R., Raju M.V.L.N., Sunder G.S. (2009). Effect of Butyric Acid on Performance, Gastrointestinal Tract Health and Carcass Characteristics in Broiler Chickens. Asian Australas. J. Anim. Sci..

